# Urban sprawl, obesity, and cancer mortality in the United States: cross-sectional analysis and methodological challenges

**DOI:** 10.1186/1476-072X-13-3

**Published:** 2014-01-06

**Authors:** David Berrigan, Zaria Tatalovich, Linda W Pickle, Reid Ewing, Rachel Ballard-Barbash

**Affiliations:** 1 Applied Research Program, Division of Cancer Control and Population Sciences, National Cancer Institute, Bethesda, MD 20892, USA; 2 Surveillance Research Program, Division of Cancer Control and Population Sciences, National Cancer Institute, Bethesda, MD 20892, USA; 3 StatNet Consulting, Gaithersburg, MD 20879, USA; 4 The University of Utah, College of Architecture and Planning, Salt Lake City, UT 84112, USA

**Keywords:** Urban sprawl, Cancer mortality, Obesity, Spatial heterogeneity, Census division, County, Health disparities, Ecological analysis

## Abstract

**Background:**

Urban sprawl has the potential to influence cancer mortality via direct and indirect effects on obesity, access to health services, physical activity, transportation choices and other correlates of sprawl and urbanization.

**Methods:**

This paper presents a cross-sectional analysis of associations between urban sprawl and cancer mortality in urban and suburban counties of the United States. This ecological analysis was designed to examine whether urban sprawl is associated with total and obesity-related cancer mortality and to what extent these associations differed in different regions of the US. A major focus of our analyses was to adequately account for spatial heterogeneity in mortality. Therefore, we fit a series of regression models, stratified by gender, successively testing for the presence of spatial heterogeneity. Our resulting models included county level variables related to race, smoking, obesity, access to health services, insurance status, socioeconomic position, and broad geographic region as well as a measure of urban sprawl and several interactions. Our most complex models also included random effects to account for any county-level spatial autocorrelation that remained unexplained by these variables.

**Results:**

Total cancer mortality rates were higher in less sprawling areas and contrary to our initial hypothesis; this was also true of obesity related cancers in six of seven U.S. regions (census divisions) where there were statistically significant associations between the sprawl index and mortality. We also found significant interactions (p < 0.05) between region and urban sprawl for total and obesity related cancer mortality in both sexes. Thus, the association between urban sprawl and cancer mortality differs in different regions of the US.

**Conclusions:**

Despite higher levels of obesity in more sprawling counties in the US, mortality from obesity related cancer was not greater in such counties. Identification of disparities in cancer mortality within and between geographic regions is an ongoing public health challenge and an opportunity for further analytical work identifying potential causes of these disparities. Future analyses of urban sprawl and health outcomes should consider exploring regional and international variation in associations between sprawl and health.

## Background

Urban areas are growing rapidly in population and land area throughout the world [1]. Much of this growth falls into a pattern characterized as ‘urban sprawl’ [2], an economic and social process associated with low residential density, segregation of land use, and automobile dependence [3]. A number of studies have reported that urban sprawl is associated with health outcomes, health behaviors, and obesity [3-7]. In cross-sectional studies, greater levels of urban sprawl are associated with increased obesity, poorer mental health, more traffic accidents, and lower levels of physical activity including walking [4,5,8-11]. These adverse associations with health could be due to increased exposure to pollution, changes in behaviors that directly influence disease risk such as smoking, sedentary time, or social behavior, or through the association of sprawl with use of health care systems [12]. Alternatively, selection of residential environments because of preference or economic constraints could also produce some of these associations. Two studies, one of childhood obesity [13] and one of walking in adults [9] report statistically significant associations between sprawl and energy balance outcomes in cross-sectional analyses but not in longitudinal ones.

Research on urban sprawl has been particularly prominent in the United States, in part because this kind of development has been very common, notably in the past 30 years [14]. However, international interest in the environmental and health consequences of sprawl is also increasing [15,16]. Independent of the analysis of urban sprawl, there is also a long and global history of examining the effect of cities and residence in urban areas on health outcomes, including cancer [17-19]. In the 19^th^ century, scrotal cancer was associated with soot exposure [20] and more recently, breast and lung cancer rates have sometimes but been reported to be higher in urban areas than rural or suburban areas [21-24]. Wilson and Chakraborty [25] highlight the lack of comparative studies across national boundaries concerning the environmental consequences of sprawl and such studies of health outcomes and health behaviors are also rare [25]. Comparative studies of urban sprawl and health could lead to insights arising from how sprawl develops and manifests itself in different countries.

Comparative studies of geographic areas can be used to examine the contributions of different risk factors to specific cancers. For example, such studies have been used to examine the relative contribution of environmental exposures and reproductive characteristics to breast cancer risk and the contributions of air pollution versus smoking prevalence to lung cancer [22,23]. There is also interest in urban–rural differences and spatial variation in stage at diagnosis. For example a study using SEER data reports that urban as opposed to rural residence is associated with stage at diagnosis for lung and colorectal cancer [26] and there is also substantial geographic variation late stage breast and colorectal cancer diagnosis [27,28]. Access to care and the distribution of race-ethnic groups are associated with county level variation in stage at diagnosis identified in some of these studies, but considerable unaccounted for variation between counties remains. Despite their limitations, ecological analyses or cross case comparisons are sometimes one of the best and most feasible ways to examine the contributions of environmental and contextual variables to health outcomes and to identify targets for focused intervention studies [28-31].

The main goal of this paper is to examine cross-sectional associations between urban sprawl and cancer mortality, accounting for spatial variation in mortality and the effects of obesity, smoking, measures of health care services and access, and socioeconomic status. Because obesity is a risk factor for cancer at a number of sites [32] and because the prevalence of obesity is greater in more sprawling regions of the US [4], we hypothesized that mortality for obesity related cancers would be higher in more sprawling areas. In addition to its association with obesity prevalence, sprawl may also have an effect on access to care. For example; increased travel time could influence access to screening and treatment facilities [33]. Thus our overall conceptual model involves a socio-ecological approach and includes urban form, modifiable risk factors, and demographic variables, as well as measures related to care. In this paper we chose to examine associations between urban sprawl and obesity-related cancer mortality. Future studies could address specific cancers, cancer related to other risk factors such as smoking, and cancer incidence.

Our analysis emphasizes the potential role of urban sprawl as an influence on obesity related cancer because of the extensive literature exploring the association between the built environment and energy balance that has appeared over the past decade [4,5,13]. However, recent reviews also suggest that the relationship between obesity and environmental variables is heterogeneous, with a lack of consistent associations across studies [7,34,35]. Few past studies of urban sprawl have examined geographic differences in the association between sprawl and health or health behaviors [12,36,37] and therefore residual spatial heterogeneity associated with such differences could account for some of the inconsistency seen in studies of the environment, health and energy balance.

A further goal of this paper is to improve upon previous approaches to the analysis of ecological data so that the effects of spatial autocorrelation are accounted for properly. Spatial autocorrelation, a form of spatial dependency, occurs when observations that are closer together in space are more similar to each other. Most studies of regional and national variation in cancer incidence and mortality do not explicitly address this issue [12,38], despite the consensus amongst spatial statisticians that it is critical to account for spatial dependency to obtain valid analytical results [39]. Instead most past studies emphasize the standard statistical assumptions of regression models such as independence, normality, and homoscedasticity of error.

In light of these goals, the specific aims of this paper are to 1) analyze associations between urban sprawl and cancer mortality in the US and to determine to what extent these associations differ in different regions of the country and 2) more comprehensively assess and address the presence of spatial autocorrelation in these mortality data. We focus on cancer mortality rather than incidence because urban sprawl has the potential to influence determinants of both cancer incidence and mortality via its relationships to risk factors such as obesity and physical activity and via its influence on factors related to cancer prevention and treatment such as transportation and the distribution of facilities.

## Methods

This cross-sectional analysis is based on 2002–2006 county level estimates of cancer mortality in the United States of America from the US Surveillance, Epidemiology and End Results (SEER) program [40], along with data originating from the National Center for Health Statistics [41]. We report results for men and women separately for cancer mortality from the 19 and 21 most common cancers in men and women respectively, and for six cancers for which obesity is a major risk factor.

**Figure 1:**
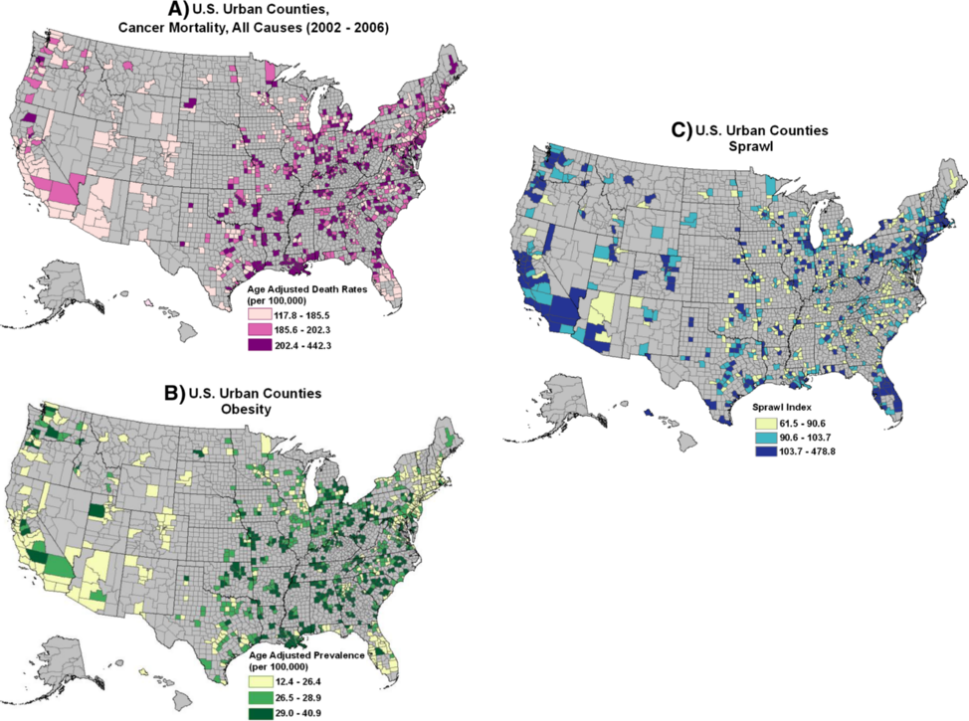
County level distributions of A) total cancer mortality (2002–2006), B) obesity (2007) and C) urban sprawl (2000) by tertiles for the continental United States.

The geographic coverage of this analysis included 935 urban and suburban counties in the US (Figure 1). These counties represent the complete set of continental US counties of interest because the concept of urban sprawl is not relevant in rural counties. They include all metropolitan statistical areas in the continental United States as defined by the US Census bureau and adjacent (‘suburban’) counties. Variables used in our analysis are summarized below and in Table 1. We also used Census Division as a variable in our analyses to explore regional variation in associations between sprawl and mortality. The US census divides the nation into nine contiguous divisions in four regions (Northeast (New England and Middle Atlantic), Midwest (East and West North Central), South (South Atlantic, East and West South Central), and West (Mountain and Pacific)). The divisions contain from three to eight states [42]. Our sample included 34 to 222 counties per division.

**Table 1:** Distributions of covariates and cancer mortality rates

Variable*	**Mean (SD)**	**Minimum**	**Maximum**
**Cancer mortality rates**			
**Females**			
All cancers	242.2 (27.5)	145.4	354.8
Obesity related	102.4 (16.0)	43.9	168.2
**Males**			
All cancers	242.0 (34.2)	123.3	361.6
Obesity related	56.4 (8.8)	5.2	96.6
**Sprawl index**			
Continental US	100 (25)	62	479
New England (N = 34)	108 (24)	79	213
Middle Atlantic (N = 89)	122 (59)	80	479
East North Central (N = 168)	97 (14)	68	171
West North Central (N = 92)	94 (14)	62	132
South Atlantic (N = 222)	95 (16)	66	188
East South Central (N = 91)	92 (11)	68	116
West South Central (N = 123)	99 (13)	74	166
Mountain (N = 50)	103 (12)	79	130
Pacific (N = 65)	113 (24)	85	258
**Other covariates**			
Obese (%)**	27.3 (3.6)	12.4	40.9
Current smokers (%)			
Female	21.9 (4.8)	7.9	36.9
Male	25.7 (5.0)	12.0	41.3
Non-hispanic white (%)	82.9 (14.3)	21.2	99.0
Non-hispanic black (%)	9.9 (12.6)	0.0	70.7
Hispanic (%)	6.7 (11.1)	0.28	94.4
American Indian/Asian PI (%)	2.5 (4.9)	0.0	55.2
MD density***	2.0 (1.8)	0.0	20.1
Hospital density***	0.021 (0.018)	0.0	0.15
Uninsured****	14.1 (4.4)	6.7	36.8
Socioeconomic position index	0.64 (0.8)	−1.67	1.89

*N = 935 for covariates, N = 934 for cancer mortality rates.

**Age adjusted% BMI ≥ 30.

***(Number/County Population)*10,000.

****Under 65 Years old.

Age-adjusted cancer mortality rates were obtained from the NCI SEER Cancer Registry (2002–2006) by averaging county values.

### Cancer mortality

County level mortality rates based on data from 2002–2006 for the 19 most common cancers in adult males (oral cavity and pharynx, esophagus, stomach, colon, liver, pancreas, larynx, lung, melanoma, bladder, kidney, brain, thyroid, Hodgkin’s lymphoma, Non-Hodgkin’s lymphoma, myeloma, leukemia, prostate and testis) and the 21 most common cancers in adult females (similar to the list for males except prostate and testis cancer are replaced with cancer of the cervix, uterus, ovary and breast) were obtained from the SEER database [43]. These cancers were selected because they were sufficiently common to allow stable mortality estimates for all the counties considered. After exploratory analyses of mortality from specific cancers, we chose to analyze mortality rates in two categories: overall cancer mortality and obesity-related cancer (colon, endometrium, kidney, esophagus, pancreas, post-menopausal breast). Obesity related cancers were categorized based on the World Cancer Research Fund [32] report on risk factors. Breast cancer mortality was divided into pre and post-menopausal on the basis of age, with breast cancer mortality in women > 50 years old assumed postmenopausal. No attempt was made to distinguish risk factors for squamous cell versus adenocarcinomas. All rates were directly age-adjusted using the 2000 U.S. population standard.

Results for one county, Madison County, MS, were excluded from regression analyses but included in the summary tables. In Madison County, both male and female cancer mortality rates for all cancers combined were > 6 SD from their respective means of the 935 counties considered. A non-profit group, the Susan G. Komen Foundation, previously identified Madison county as having very high cancer mortality rates and the Centers for Disease Control and Prevention (CDC) has attributed these high rates to the presence of a hospice serving all of central Mississippi [44]. Discussion with SEER staff yielded no further clues as to alternative explanations as to why this county had such high rates.

### Sprawl index

The urban sprawl index used here is a composite measure of features of urban form related to population density and street accessibility [4]. In brief, the index, which arrays counties from least to most sprawling, has its highest values in urban counties such as the four urban boroughs of New York City or San Francisco County, and its lowest values in the suburbs of sprawling metropolitan regions throughout the country [4,45]. Because of this inverse scaling, some readers may find it easier to think of this as a ‘compactness’ index [46]. Jackson County, adjacent to Topeka KS, had the greatest level of sprawl in this data set. The index is based on six variables extracted from US census data - 1) Gross population density in persons per square mile, 2) Percentage of population living at densities less than 1,500 persons per square mile (low suburban density), 3) Percentage of population living at densities greater than 12,500 persons per square mile (transit-oriented urban density, 4) net urban density (the ratio of population to urbanized land area from US census data), 5) Average block size in square miles, and 6) Percentage of small blocks, roughly 500 feet on a side. The first four of these variables are related to population density and the remaining two variables are related to block characteristics and street connectivity. For this paper, the sprawl index was calculated based on US Census data from 2000.

### Obesity

County level obesity prevalence estimates are based on self reported height and weight summarized by the CDC. Estimates for each county are based on Bayesian analysis combining information from the US Behavioral Risk Factor Surveillance System (BRFSS) and the US Census [47,48].

### Smoking

Estimates of county level current smoking prevalence for men and women during 2000–2003 were downloaded from the National Cancer Institute (NCI) Division of Cancer Control and Population Sciences (DCCPS) small area estimates web page [49]. These prevalence estimates were calculated using information from the National Health Interview Survey (NHIS) and the BRFSS using a model-based estimation procedure [50]. Note that this estimation procedure uses covariates such as race/ethnicity, age, and education levels. Some of these covariates are also used in our analysis.

### Race/ethnicity data

County level prevalence of self-identified Non-Hispanic White, Non-Hispanic Black, Hispanic and American Indian, Asian, and Pacific Islander (AIAPI) race/ethnic groups were obtained from the 2000 US Census. Prevalence of American Indian, Asian and Pacific Islander were combined into a single category. Analyses that excluded American Indians gave qualitatively similar results (not shown).

### Socioeconomic position index

We calculated a version of the Krieger 2002 Socioeconomic Position Index (SEP) index [51] for each county. The SEP represents a summary deprivation measure consisting of a standardized z score combining data on percentage working class, unemployment, percentage below US poverty line, low education, expensive homes, and median household income. Our version of the index was based on a single principal component from the Principal Components Analysis (PCA) of the six variables considered. Higher values of the index indicate higher socioeconomic position. For example, the five counties with the lowest values of the SEP are Tunica MS, Hidalgo, Cameron, and Webb Counties (TX), and Terrell County (GA) whereas the five counties with the highest values of SEP are Douglas CO, Loudon VA, Hamilton IN, Howard MD, and Johnson County KS.

### Access and availability of care

Information concerning physician density, hospital density, and health insurance coverage were used as proxies for access and availability to health care resources [33]. These variables were extracted from the 2008 release of the Area Resource File (ARF) for the year 2002 [52]. Total active non-federal and federal physicians and number of hospitals per 1000 people in the county were selected for analysis. Prevalence of uninsured aged 0–64 was also calculated from ARF health care and Census population data. Because prevalence of health insurance coverage was positively associated with cancer mortality, contrary to many results at the individual level [53], we also extracted median age of adults from census data in order to determine if residual variation in age structure accounted for the ecological association of insurance prevalence and cancer mortality (It did not, see below).

### Statistical analysis

We used SAS JMP and R to initially examine the association between cancer mortality and the independent variables listed above and to check that the data met the assumptions required of the proposed linear regression models [54,55]. The age-adjusted mortality rates were approximately normally distributed so no data transformation of the rates was necessary. Variables relating to race/ethnicity, physician (MD) density, hospital density, and the sprawl index were natural log transformed to improve the linearity of their associations with mortality. Additionally, cancer mortality rates were weighted by county population size in order to stabilize their variances for large and small counties, an assumption also required by the subsequent models. Separate models were fit for males and females and for both of the cancer groupings.

Our overall approach involved fitting an increasingly complex series of normal linear regression models to the age-adjusted mortality rates and then examining to what extent adding more explanatory covariates, interactions and spatial autocorrelation accounted for the observed variation in mortality rates. Important two way interactions between the main effect covariates were identified for inclusion in the models using the Elastic Net approach, a weighted average of the least absolute shrinkage and selection operator (LASSO) and Ridge regression variable selection methods [56]. This method has been shown to be more robust than conventional stepwise regression variable selection methods and overcomes problems of selection from among highly correlated variables by either the LASSO or Ridge methods alone [56,57]. Interactions from this initial variable selection process were retained in the subsequent regression models only if they were significant (p value < 0.05) Because the Elastic Net method evaluates groups of the covariates and interactions, unlike stepwise regression methods, no further adjustment for multiple comparisons was needed. Note that final models included different interactions for the different gender and cancer groupings.

As noted above, our main study aim involved understanding the relationship between urban sprawl and cancer mortality. Therefore, we began by examining simple models estimating the relationship between mortality and urban sprawl without any other covariates. For each of the four groups (Overall and obesity related cancer mortality by gender) six models were fit and examined for the presence or absence of spatial auto-correlation. These models included successively more variables related to cancer causes and spatial structure: Model 1: ln sprawl, Model 2: ln Sprawl + demographic and other covariates, Model 3: ln Sprawl + demographic and other covariates + Census Division, Model 4: ln Sprawl + demographic and other covariates + interactions + Census Division, Model 5: ln Sprawl + demographic and other covariates + interactions + Census Division + random effects to account for county-level spatial autocorrelation, Model 6: ln Sprawl + demographic and other covariates + interactions + Census Division + County random effects + ln Sprawl*Census Division interaction.

All models were implemented using SAS PROC GLIMMIX [58] by pseudo-likelihood estimation with population weights. For the spatial autocorrelation models, a spatial variance component was included as a random effect on the linear predictor (in SAS terms, a G-side effect). This parameter was estimated by a spline smoothing algorithm over a regular grid that spanned the continental US, i.e., with spatial autocorrelation based on distances between all pair-wise county locations. Location was defined by latitude and longitude of the county geographic centroids. Broad regional spatial patterns were assessed by inclusion of indicator variables for Census Divisions as fixed effects in the models.

Because there is no single statistic, such as a likelihood ratio statistic, that adequately measures the goodness of fit of spatial mixed effects models, we examined fit using several different techniques [39,59]. A standard set of residual plots was generated for each model and examined for model fit and consistency with distributional assumptions. We also examined overall fit of the models by tabulating pseudo R^2^ and Akaike’s Information Criterion (AIC) values. The pseudo R^2^ statistic measures the proportion of total variance explained by the model (1-(sum of squared errors)/(total sum of squares)). AIC is a measure of the regression sum of squares that includes a penalty for every added covariate [60]. The “best” of the 6 models is the one with the highest R^2^ and/or the lowest AIC value.

Because one of our objectives was to explore the extent to which these models accounted for spatial patterns and autocorrelation in mortality rates, we also calculated Moran’s I statistic for the standardized residuals of the six models described above using a one-sided permutation test [39]. This statistic does not account for variation in rates due to population differences, so we used it to rank the degree to which each model reduced the spatial patterns apparent in the original data. Successive efforts to account for spatial effects involved models with a) our complete set of covariates to explain spatial patterns at the county level, b) inclusion of US Census Division to explain broader regional patterns, and c) spatial random effects to account for any remaining spatial autocorrelation, i.e., the tendency of mortality rates for neighboring counties to be similar. All of these three elements were required to adequately account for the spatial patterns and autocorrelation in the data. We included the interaction term between our measure of sprawl and Census Division in our final models because it represents an explicit test of regional heterogeneity in the association between sprawl and mortality.

## Results

### Descriptive statistics

Characteristics of the 935 counties studied here and mortality rates for the cancers examined are presented in Table 1. Note that the mean and standard deviation of the sprawl (‘compactness’) index (100, 25) reflect the standardization procedure used to develop the index [4]. Obesity prevalence and the sprawl index used here were negatively associated (r = −0.36), this indicates lower prevalence of obesity in less sprawling areas because a higher sprawl index corresponds to a denser more urbanized county. Overall, US counties exhibit a wide range of values for all the variables used in our analyses.

The twelve variables considered in this analysis are mostly weakly correlated with one another. The strongest correlation (0.79), between male and female smoking prevalence, represents two variables that are not included in the same models. The second strongest correlation (−0.71) was between proportions of non-Hispanic whites (Treated as the reference group in our models) and blacks. There were statistically significant positive correlations from 0.52- 0.62 between the prevalence of obesity and smoking in men and women, between the log transformed sprawl index and the proportion of American Indian, Asian and Pacific Islanders (AIAPI) and physician density and between the proportion Hispanic and AIAPI. Thus, the proportion of AIAPI was greater in more urban areas. There were significant negative correlations (−0.52—0.56) between prevalence of smoking in females and the proportion Hispanic and AIAPI and between current smoking in males and the SEP. The remaining 66 correlations were not statistically significant. The modest correlations among most of the variables suggest that we have identified a robust set of county level characteristics.

Cancer mortality rates for the four groupings we examined showed substantial variation at the county level (Table 1). The range of mortality rates for cancer related to obesity varied by four-fold in females and by almost 20-fold in males among counties and rates for overall cancer mortality varied by 2–3 fold. Geographic distributions of the urban sprawl index, overall cancer mortality for both sexes combined and the prevalence of obesity are illustrated in Figure 1A-C. Note that multiple urban and suburban counties are found in every state in the continental U.S.

### Spatial autocorrelation

To determine if our simple models accounted for spatial autocorrelation in cancer mortality rates across these 935 urban and suburban counties, we calculated Moran’s I statistic for a series of models for both genders and the two cancer groups of interest (Table 2). Analysis of residuals from the simplest models (ln Sprawl only) for all four of our cancer mortality groupings and two of the more complex models for females showed highly significant unexplained spatial autocorrelation (Table 2). The addition of random effects to capture spatial autocorrelation of rates at the county level reduced all Moran’s I values to non-significant levels (Table 2, models 5 & 6). Furthermore, the spatial variance component parameters measuring autocorrelation across counties were significant in the most complex models for overall cancer among both men and women and obesity-related cancers among men. Together these results suggest that the simple fixed effects models did not adequately account for spatial patterns in the residuals. In subsequent models we included census divisions to measure broad regional effects and a random effect for spatial autocorrelation between counties. After inclusion of these variables in the models there is little spatial variation in mortality rates that is not explained by the specific demographic, risk, and health care related factors and location effects included in the more complex analyses. This allows us to perform a robust analysis of associations between urban sprawl and cancer mortality.

**Table 2:** Moran’s I test for spatial autocorrelation for alternative models

**Covariates included in models**	**All cancers**				**Obesity related cancers**			
	**Male**		**Female**		**Male**		**Female**	
	**I**	**p**	**I**	**p**	**I**	**p**	**I**	**p**
1. ln sprawl	**0.099**	≤0.0001	**0.049**	≤0.0001	**0.067**	≤0.0001	**0.039**	≤0.0001
2. ln sprawl + other covariates	−0.005	0.999	**0.004**	0.005	−0.006	0.999	−0.0001	0.154
3. ln sprawl + other covariates + census divisions	−0.003	0.992	0.001	0.051	−0.001	0.487	−0.0006	0.231
4. ln sprawl + other covariates + census divisions								
+ selected interactions	−0.003	0.986	**0.002**	0.022	−0.002	0.669	**0.002**	0.03
5. ln sprawl + other covariates + census divisions								
+ selected interactions + county centroids	−0.002	0.91	−0.002	0.588	−0.002	0.687	−0.001	0.436
6. ln sprawl + other covariates + census divisions								
+ selected interactions + county centroids								
+ ln sprawl * census division interaction	−0.002	0.827	−0.002	0.792	−0.002	0.687	−0.002	0.767

Values in bold are associated with significant Moran’s I, p < 0.05.

### Model results

Preliminary analyses indicated that associations between mortality and the sprawl index varied significantly by Census Division. Therefore, we chose to include this interaction term (ln sprawl*Census Division) in the final random effects models presented here. Coefficients for the main effects of the nine covariates in addition to urban sprawl included in each model are given in Table 3 and coefficients for Census Division and ln sprawl*Census Division are presented in Table 4. Because the interactions between census division and natural log transformed sprawl index were significant, we do not report overall coefficients for the sprawl index.

**Table 3:** Random effects model results

	**All cancers**		**Obesity related**	
	**Coefficients**	**SE**	**Coefficients**	**SE**
Females**				
Current smoking	224.21*	20.2	**85.85**	12.75
Ln hispanic	0.55	1.1	−0.22	0.58
Ln NH black	**10.50**	1.0	**6.62**	0.62
Ln AIAPI	**−3.31**	1.2	−1.42	0.71
Ln MD density	5.35	1.5	1.00	0.88
Ln hospital density	−2.73	1.9	−1.77	1.11
Obesity	0.04	0.2	−0.02	0.16
SEP index	**5.85**	2.3	5.30	2.63
Uninsured	**−60.89**	24.1	37.52	31.85
AIC	8238	7124		
Gener. chi-square/df	49474989	16970961		
Males				
Current smoking	**207.90**	20.95	**34.96**	6.64
Ln hispanic	0.06	1.13	−0.33	0.35
Ln NH black	**4.02**	1.07	**1.32**	0.33
Ln AIAPI	**−5.22**	1.26	**−1.31**	0.40
Ln MD density	4.48	5.05	−0.37	1.62
Ln hospital density	3.0	1.89	0.31	0.61
Obesity	**1.26**	0.29	**0.22**	0.09
SEP index	3.84	2.56	0.76	0.78
Uninsured	**−140.89**	24.06	**−35.24**	7.60
AIC	8165	6125		
Gener. chi-square/df	45146764	4801908		

*Coefficients in bold are significantly different from zero, p ≤ 0.05.

**Interactions Included in the models were statistically significant with p ≤ 0.01. For males: All cancers; MD*SEP, SEP*CD, MD* CD, and 2) Obesity related cancers; MD*SEP, SEP*CD, MD*CD, and for females: 1) All cancers: MD*SEP, SEP*CD and 2) Obesity related cancers: Smoking*SEP, Black*SEP, MD*SEP, SEP*CD, Uninsured*CD, Note that the variables name above are abbreviated MD = Physician density, CD = Census Division, SEP = Socioeconomic Position, NH = Non-Hispanic, and ‘Ln’ (natural log) has been removed.

Results for Census Division (CD) and CD*ln Sprawl are given in Table 4.

**Table 4:** **Model results for census divisions and sprawl, controlling for factors listed in Table**3

	**All cancers**		**Obesity related**	
Census division*	Coefficients**	**SE**	**Coefficients**	**SE**
**Females**				
**Intercepts**	346.5	79.1	126.2	26.6
*Census divisions*				
1 New England	**−250.4**	74.5	**−95.9**	44.0
2 Mid-Atlantic	−4.0	44.3	−26.1	23.0
3 East North Central	**−265.3**	53.9	**−97.6**	29.0
4 West North Central	−109.1	82.8	−45.6	50.3
5 South Atlantic	**−199.0**	56.4	**−98.8**	30.3
6 East South Central	**−307.4**	105.2	**−208.6**	66.3
7 West South Central	**−239.8**	75.1	**−154.5**	42.1
8 Mountain	39.4	118.9	13.2	65.5
9 Pacific (Reference)	0			0
Ln Sprawl*Census Division				
1 New England	13.8	13.5	12.4	9.4
2 Mid-Atlantic	**−32.6**	5.7	−2.3	3.1
3 East North Central	**23.7**	9.1	**11.1**	5.2
4 West North Central	−7.6	16.6	0.4	9.7
5 South Atlantic	8.0	9.9	**14.0**	5.4
6 East South Central	32.1	22.0	**35.9**	13.4
7 West South Central	21.7	14.7	**27.0**	8.8
8 Mountain	−41.4	24.1	−15.5	13.5
9 Pacific	**−29.9**	8.3	−8.4	4.4
**Males**				
**Intercepts**	209.7	128.5	14.3	27.9
*Census divisions*				
1 New England	**−222.9**	101.3	−39.2	30.6
2 Mid-Atlantic	**121.8**	59.3	**55.8**	18.1
3 East North Central	**−197.7**	68.9	−**53.4**	21.1
4 West North Central	−183.1	104.4	−6.7	32.9
5 South Atlantic	−68.8	70.3	7.8	21.9
6 East South Central	**−484.2**	151.6	**−192.2**	48.2
7 West South Central	15.9	97.5	−22.7	30.5
8 Mountain	−78.4	126.5	−23.9	39.4
9 Pacific (Reference)	0		0	
Ln Sprawl* Census division				
1 New England	21.1	19.02	10.9	5.7
2 Mid-Atlantic	**−50.9**	6.6	**−8.4**	2.1
3 East North Central	19.9	10.8	**15.4**	3.4
4 West North Central	20.3	20.8	5.8	6.6
5 South Atlantic	−7.4	11.2	1.6	3.6
6 East South Central	**87.4**	31.9	**46.5**	10.2
7 West South Central	−18.7	18.7	10.0	5.9
8 Mountain	0.5	24.9	10.7	7.8
9 Pacific	−11.5	11.4	7.0	3.5

*States contained in the US Census Divisions are available at [42].

**Coefficients in bold are significantly different from zero, p ≤ 0.05.

**Figure 2:**
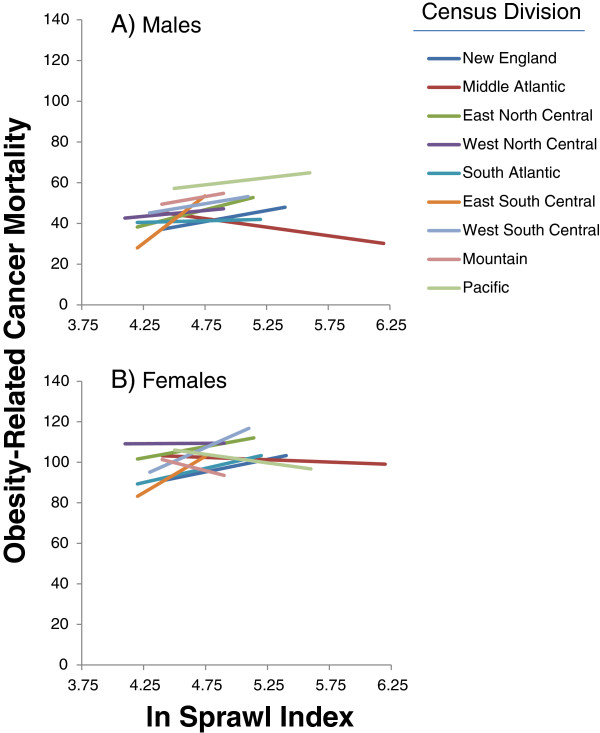
**Model based estimates of the association between natural log-transformed sprawl (‘compactness’) index and obesity-related cancer mortality for males (A) and females (B) from data for 934 urban and suburban counties in the United States.** States included in the census divisions are available at the US Census [42]. The length of each regression line reflects the range of observed values in the Divisions (Table 1).

We found significant interactions between the natural log (ln) transformed sprawl index and Census Division for all four groupings (Table 4), in other words, the slope of the association between this urban sprawl index and cancer mortality rate differed in different parts of the US. Predicted rates relating mortality from obesity related cancers and the urban sprawl index are given in Figure 2. For females, the interaction terms for census division and ln sprawl for obesity related cancer mortality were statistically significant for the East North Central States, South Atlantic States, and East and West South Central States. For males, the interaction term was significant in the Mid-Atlantic, East North Central, and East South Central Census Divisions and marginally significant in the Pacific Division. Six of the seven statistically significant interactions were positive, with a negative association in males in the Mid-Atlantic division. In other words, obesity related cancer mortality rates were generally higher in less sprawling counties (Figure 2A,B).

Current smoking was significantly and positively associated with mortality for all four groups examined (Table 4). The associations were strongest for total cancer mortality. Total cancer mortality for both genders was higher in counties with greater prevalence of non-Hispanic blacks, higher prevalence of obesity, and lower where prevalence of American Indians and Asian and Pacific Islanders was greater. A higher SEP was associated with total cancer mortality in females, but not in males. For obesity related cancers, physician and hospital density were negatively associated with cancer mortality in males but not in females. Prevalence of uninsured under age 64 was negatively correlated with all cancer mortality in both sexes. We discuss this counterintuitive result below.

Interactions included in our final models (Table 3) were all statistically significant (p ≤ 0.01). For males, a total of six interactions were included across the four cancer groupings, all including Census Division, physician density or both. For females, seven interactions were included; all seven of these included Census Division, SEP or both. Because our main interest here is in urban sprawl, we do not present detailed results for these interactions; nevertheless, they further highlight the presence of regional heterogeneity in cancer mortality rates at the Census Division level.

To assess goodness of fit, we report AIC and pseudo R^2^ values in Table 5. AIC values were lowest in our final models in all cases and R^2^ values were highest or nearly highest (Table 5). Thus, all measures of model fit, including Moran’s I statistic, support our choice of models with Census Division, its interaction with sprawl and spatial autocorrelation random effects included in the models.

**Table 5:** Model comparisons by cancer group

	**All cancers**		**Obesity related**	
	AIC*	**R**^**2**^	**AIC**	**R**^**2**^
**Females**				
1. ln_sprawl	9298.40	0.0069	7968.52	0.0034
2. ln_sprawl + other covariates	8735.42	0.1953	7635.26	0.0749
3. ln_sprawl + other covariates	8599.35	0.1886	7518.19	0.0831
+ census divisions				
4. ln_sprawl + other covariates	8517.52	0.1813	7303.8	0.1059
+ census divisions + interactions**				
5. ln_sprawl + other covariates	8351.39	0.2764	7207.87	0.1318
+ census divisions + interactions				
+ spatial autocorrelation				
6. ln_sprawl + other covariates	8238.44	0.2769	7124.72	0.1379
+ census divisions + interactions				
+ spatial autocorrelation +				
ln_sprawl * census division interaction				
**Males**				
1. ln_sprawl	9678.69	0.1331	6977.84	−0.0027
2. ln_sprawl + other covariates	8748.26	0.5882	6558.02	0.1691
3. ln_sprawl + other covariates	8628.94	0.6149	6431.43	0.2227
+ census divisions				
4. ln_sprawl + other covariates	8511.83	0.6125	6367.28	0.2346
+ census divisions + interactions**				
5. ln_sprawl + other covariates	8287.08	0.6878	6238.89	0.2948
+ census divisions + interactions				
+ spatial autocorrelation				
6. ln_sprawl + other covariates	8165.92	0.6913	6125.37	0.2994
+ census divisions + interactions				
+ spatial autocorrelation +				
ln_sprawl * census division interaction				

*Akaike information criterion.

**Interactions included are described in Table 3 and the text.

## Discussion

This cross-sectional ecological analysis of urban sprawl and cancer mortality was designed to examine associations between cancer mortality and urban sprawl and to determine if such associations differed in different regions of the United States. The study had two main results. **First,** obesity related cancer mortality rates are either higher in counties with less sprawling urban form or unrelated to sprawl. This result extends a recent analysis by Fan and Song [12] that reported positive associations between total cancer mortality and sprawl in a subset of the suburban counties examined here and also reported higher mortality associated with cancer in urban compared to suburban counties. Considerable literature has also examined rural–urban differences in mortality, with mixed results [24,61]. Some studies have also reported risk of diagnosis at later stages of cancer is higher in urban compared to rural or suburban areas [19,26]. More specific measures of factors varying along urban-suburban and urban–rural gradients such as the sprawl (‘compactness’) index examined in this paper could help clarify how mortality and stage at diagnosis are associated with features of such environments.

**Second**, the association between urban sprawl and obesity related cancer mortality differs in different regions of the United States, with more positive associations in the southern part of the country and the Great Lakes area for females and the central part of the US for males (Table 3; Figure 2). Obesity related cancer mortality was higher in more sprawling counties in the Mid-Atlantic States for males. The least sprawling county in the US (New York County NY) has a sprawl index of ~480 (natural log = 6.17) whereas Montgomery County, MD, adjacent to Washington DC, a county with an intermediate level of sprawl, has a sprawl index of ~120 (natural log = 4.79). The most sprawling counties in the US, have indices of ~ 70 (natural log = 4.25). Thus, large differences in urban form are associated with modest differences in cancer mortality.

Nevertheless, these associations between sprawl and cancer mortality could have public health significance because of the large number of people involved. Greater than 70% of the US population lives in the urban and suburban counties analyzed here. The association between obesity related cancers and sprawl was not qualitatively altered by the inclusion of county level obesity prevalence in the model despite the presence of cross-sectional associations between obesity and urban sprawl found in several past studies [4,5,7]. This suggests that the elevated mortality rates observed in less sprawling areas in the south and Great Lakes regions could be caused by social, medical care related, or risk factors not adequately accounted for in our models rather than obesity prevalence.

Comparative studies of diverse countries concerning the health and environmental consequences of sprawl are lacking [25]. Recent studies have reported that active transportation is more common amongst Canadian 12–15 year olds residing in more sprawling areas [15] and in a large study of Portuguese adults population density was not associated with physical activity or BMI [62]. In both the US and in other countries, analyses of rural–urban differences in cancer mortality have had mixed results [21-24,61,63]. Careful comparative studies of urban sprawl and cancer incidence and sprawl across diverse regions and countries could provide further insight into associations of specific features of urban form and health outcomes.

Strengths of this analysis include that we make a rigorous attempt to account for spatial variation in cancer mortality and in the distribution of cancer risk factors at the county level across the United States. This effort involves successive elaboration of our regression models plus allowing for possible spatial autocorrelation among neighboring counties. We argue that this is important, because of the proliferation of ecological models that include geographic variables, but fail to test whether or not they account for spatial variation in the dependent variables of interest [12,38].

Our finding that the association between sprawl and cancer mortality varies by region suggests that future analyses of the health effects of sprawl should formally include analysis of regional differences. Most past studies of sprawl and energy balance have not focused on regional variation [3-5,9,64]. Recently, Troped et al. [36] reported regional difference in associations between perceived built environment and physical activity but associations between perceived crime and activity did not appear to differ by region [36]. The analyses in our paper adjust for the effects of demographic, economic and health care system related variables. Additional explanations for the regional heterogeneity observed in relations between sprawl, mortality and behavior could also be sought through consideration of regional differences in transportation systems, built environment aspects of sprawl, climate related factors, or inadequate control for residual confounding by risk factors included in our models. For example, counties classified as urban or suburban in the western United States included some very large counties such as Coconino, Yuma and Pima Counties in Arizona, San Bernardino County in California, and San Juan County in New Mexico. Additionally, suburban counties in some areas of the US are almost entirely residential, whereas in the Midwest and Western United States, suburban counties may have a large amount of agricultural or undeveloped land. Nevertheless, our results suggest that future geospatial analyses of cancer mortality and urbanicity across the US should attempt to account for regional variation in such associations and further explore sources of such variation.

### Insurance coverage by county

One ecological puzzle emerged from this analysis. We found that the prevalence of lack of insurance coverage in subjects under age 65 was negatively associated with total cancer mortality rate in both males and females. In other words, cancer mortality rates were lower in counties where prevalence of insurance coverage was lower. Two recent analyses using SEER mortality data concerning urological cancers (Prostate, Bladder, and Kidney) also report negative associations between prevalence of uninsured and cancer mortality [38,65]. These results contrast with a large body of evidence indicating that insurance coverage decreases mortality rate at the individual level [53]. Incomplete adjustment for age does not seem to account for this observation; inclusion of median age in our regression models did not alter these findings, although more detailed analysis of the potential effects of age structure may be necessary since everyone over 65 in the US is covered by Medicare health insurance.

Heterogeneity in age, health and insurance coverage among Hispanics may also account for the observed association between lack of insurance coverage and lower cancer mortality at the county level despite the inclusion of prevalence of Hispanic race/ethnicity in our regression models. Lack of insurance coverage and Hispanic race ethnicity are correlated at the county level in this data set and the counties with high prevalence of uninsured and lower cancer mortality are among the counties with the highest proportion Hispanic. The Hispanic population also has a younger age distribution that non-Hispanic Whites and Blacks. However, the interactions between prevalence of insurance and Hispanic race/ethnicity were not significant, thus the associations observed here occurred independent of each other. Furthermore, these counties span the whole range of socioeconomic conditions as measured by the SEP index. More attention to this ecological puzzle may be warranted in light of the changes occurring in US health care policy.

### Study weaknesses

A significant weakness of this study is the reliance on cross sectional data. A cross sectional analysis, such as this one, cannot be used to draw causal inferences concerning dependent variables. Nevertheless, we think that our analysis is useful in that it draws attention to regional heterogeneity in the association between sprawl and cancer mortality and supports a renewed focus on the higher cancer mortality rates observed in urban areas. Additionally, urban form changes slowly, therefore cross sectional studies do have the unique ability to allow examination of large differences in urban form.

Our analysis lacks information concerning two important cancer related exposures. First, environmental exposures to pollutants; such data are difficult to obtain in a uniform format for this complete sample of urban and suburban US counties. Exposure data might also be most relevant to analyses of specific cancers and we hope the analytic framework developed here will foster interest in pursuing such analyses. Second, data concerning physical activity and sedentary time. Sedentary behavior and physical activity are also associated with cancer incidence and mortality at several sites [32]. Physical inactivity data based on self-report are available at the county level [66]. However, we chose not to include this potential covariate in part because it is highly correlated with obesity prevalence, and in part because of the limits of self reported PA data [67]. Self report of obesity and smoking behavior are also subject to measurement error, but these errors are better understood than measurement error in physical activity [68,69]. Lack of information about physical activity and environmental pollutants could result in omitted variable bias if they are part of a causal connection between urban sprawl and cancer mortality. Such omitted variable bias or unmeasured confounders can influence regression results [70].

A potential weakness, but a difficult one to evaluate, involves the fact that our county level estimates of BMI and smoking are not actual measurements but the results of statistical models that include county demographic information also included in our models. This approach has the potential to increase the correlations between some of the explanatory variables in our models. However, because the covariates used to improve estimates of county level obesity and smoking prevalence are not strongly correlated with the other demographic variables used in our models, this problem may be a small one. Nevertheless, our overall analysis would be more robust if we could use estimates from data collected with sufficient accuracy in the field or, if that is not possible, values that are not contingent on these demographic variables [71,72].

Additional challenges involve the level of spatial aggregation examined here and our use of a random effects regression framework as opposed to a hierarchical linear modeling approach [73] with levels of variance estimated for several nested geographic areas. It is certainly not obvious that county is the appropriate level of spatial aggregation in which to examine cancer mortality rates in relation to urban sprawl and the various covariates considered in this analysis. Multilevel analysis that accounted for individual as well as regional characteristics could also be informative, for example by helping to account for the unexpected direction of the association between insurance coverage and mortality observed in this analysis. Such analysis might also shed further light on the observation that mortality rates for cancer were higher in more urban areas. Analyses at different levels of geographic aggregation and analyses incorporating individual, as well as aggregate, variables are possible. However, at present, US cancer mortality statistics are publicly available only at the county level and death certificates do not include information on diverse cancer related covariates at the individual level [43]. Furthermore, access to the geocoded mortality data needed for such analyses requires negotiation with each SEER registry site and with the CDC. Thus significant barriers remain to more complete and multi-level analysis of mortality data.

## Conclusions

Urban sprawl appears to have diverse negative health and environmental consequences. However, this study demonstrates that the cross-sectional associations between sprawl and mortality differ in different regions of the Unites States. Our results and those of other recent papers on this topic [12,36] should encourage a more nuanced view of the association between sprawl and health as well as future studies that explore regional differences in the effects of urban sprawl. More longitudinal studies of sprawl and health [9,13] as well as multilevel analyses, regardless of study design [11,37,74] will continue to clarify the potential for causal associations between urban form and health. There are many existing cohort studies that would allow such analyses.

Renewed attention to the causes of elevated cancer mortality rates in urban areas could help address disparities in cancer mortality. Preliminary results suggest that there are also regional differences in the association between urban sprawl and tobacco related cancer mortality. Thus, future studies could examine tobacco-related (oral cavity and pharynx, esophagus, larynx, lung, bladder) cancers using a similar approach to the one outlined here. Analyses of associations between sprawl and cancer incidence are also a logical next step in an effort to identify specific factors increasing cancer mortality in less sprawling urban and suburban counties both in the US and potentially in other countries where sprawl has emerged as a common pattern of development.

## Competing interests

The authors declare that they have no competing interests.

## Authors’ contributions

DB, ZT, LP, RE, and RBB conceived of the project. RE developed the sprawl index. DB, ZT, and LP carried out the statistical analysis. DB wrote the first draft of the paper. All authors reviewed and approved the final version of the paper.
